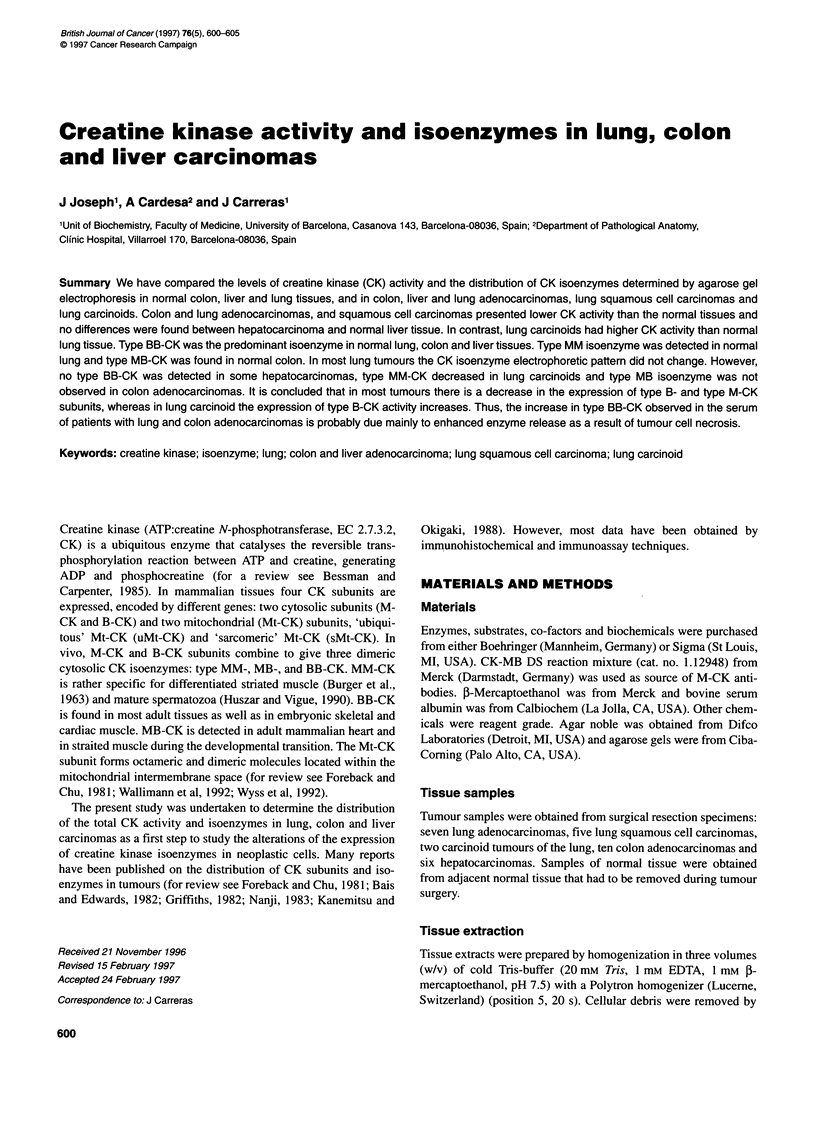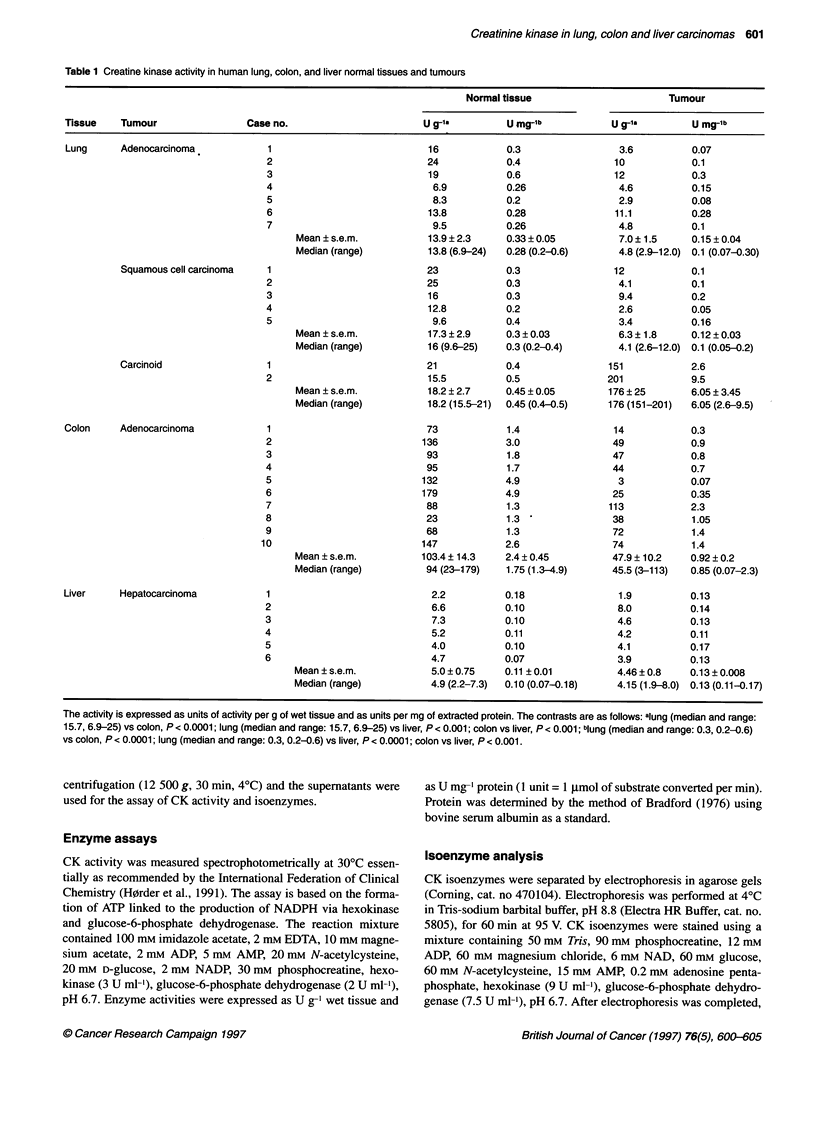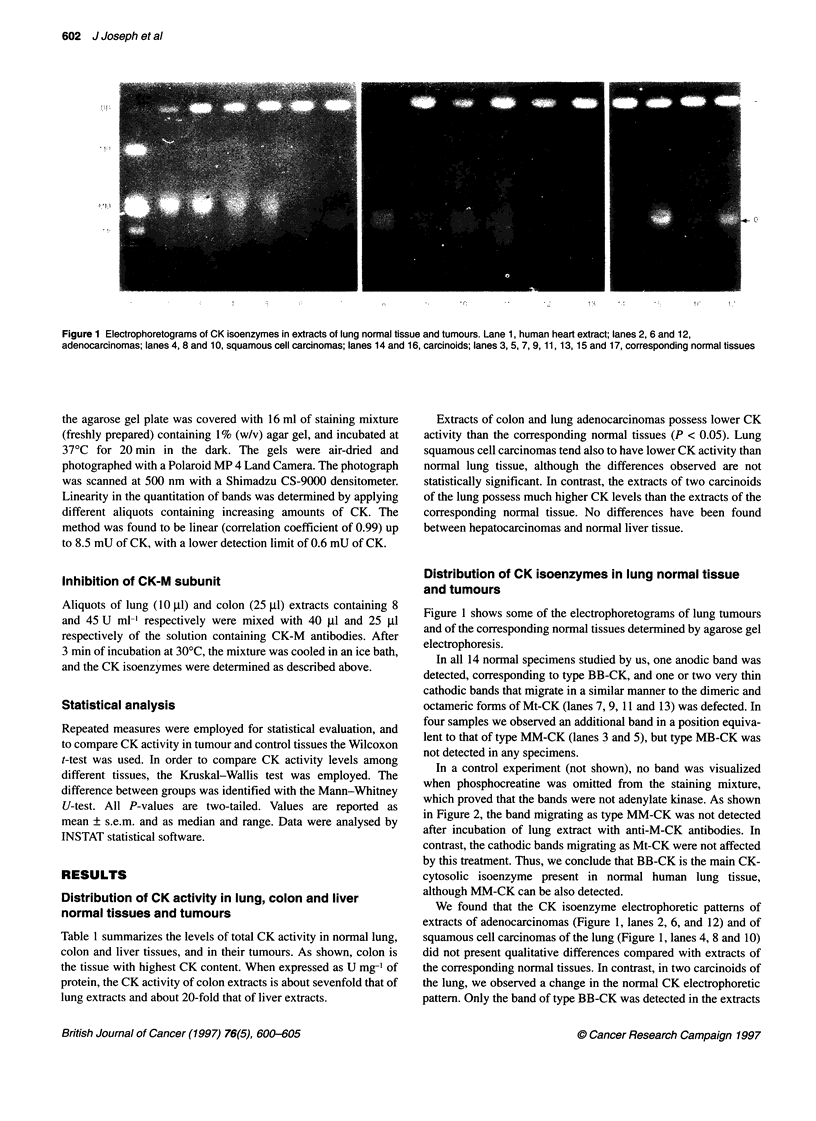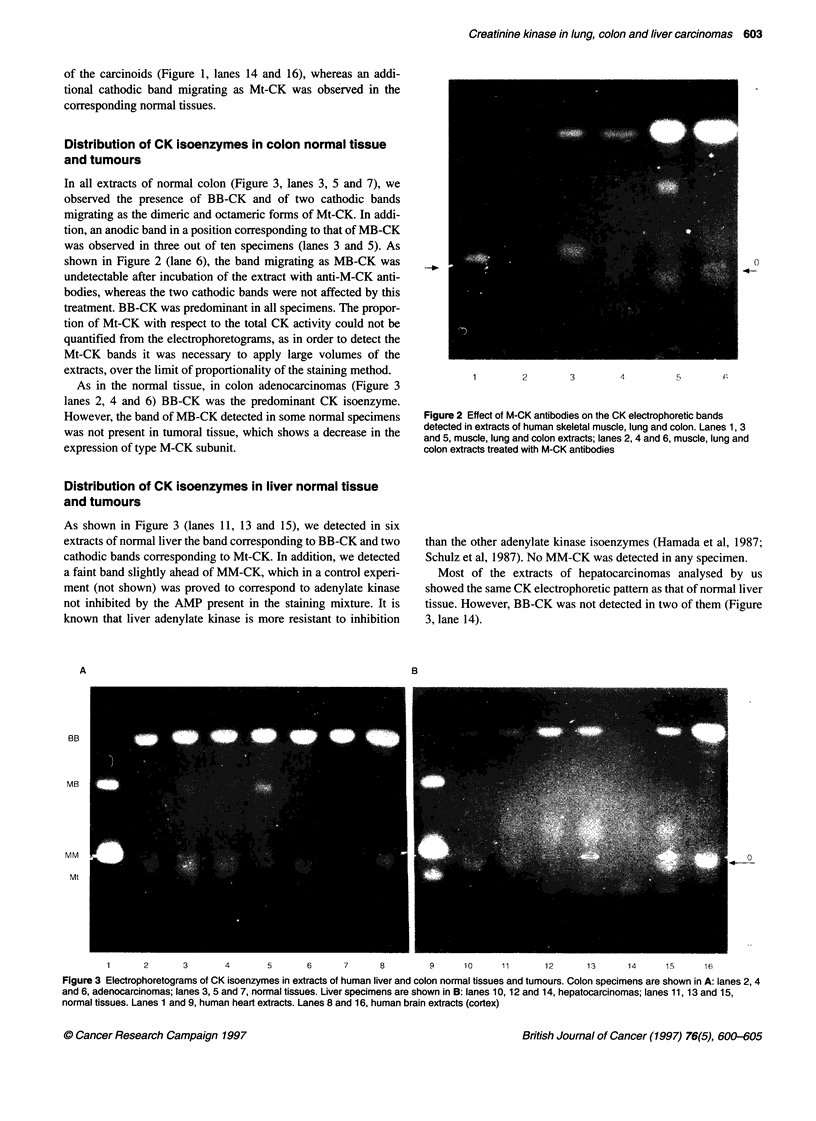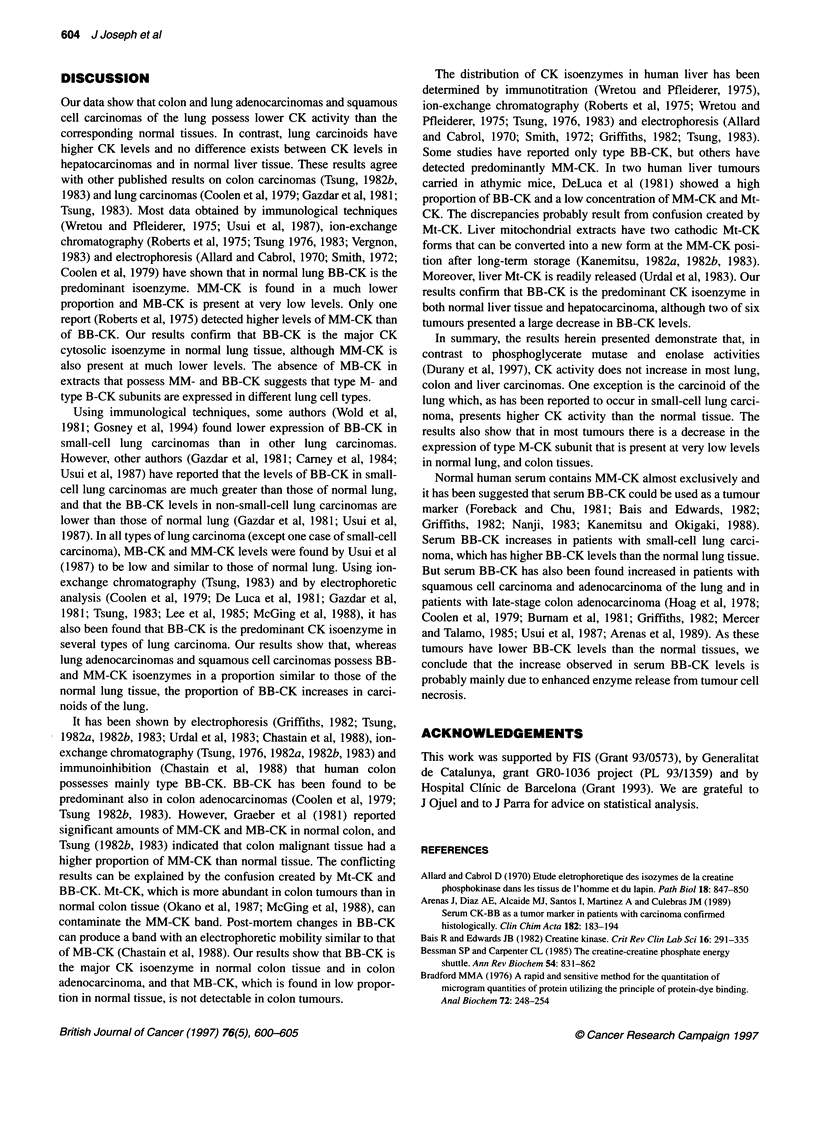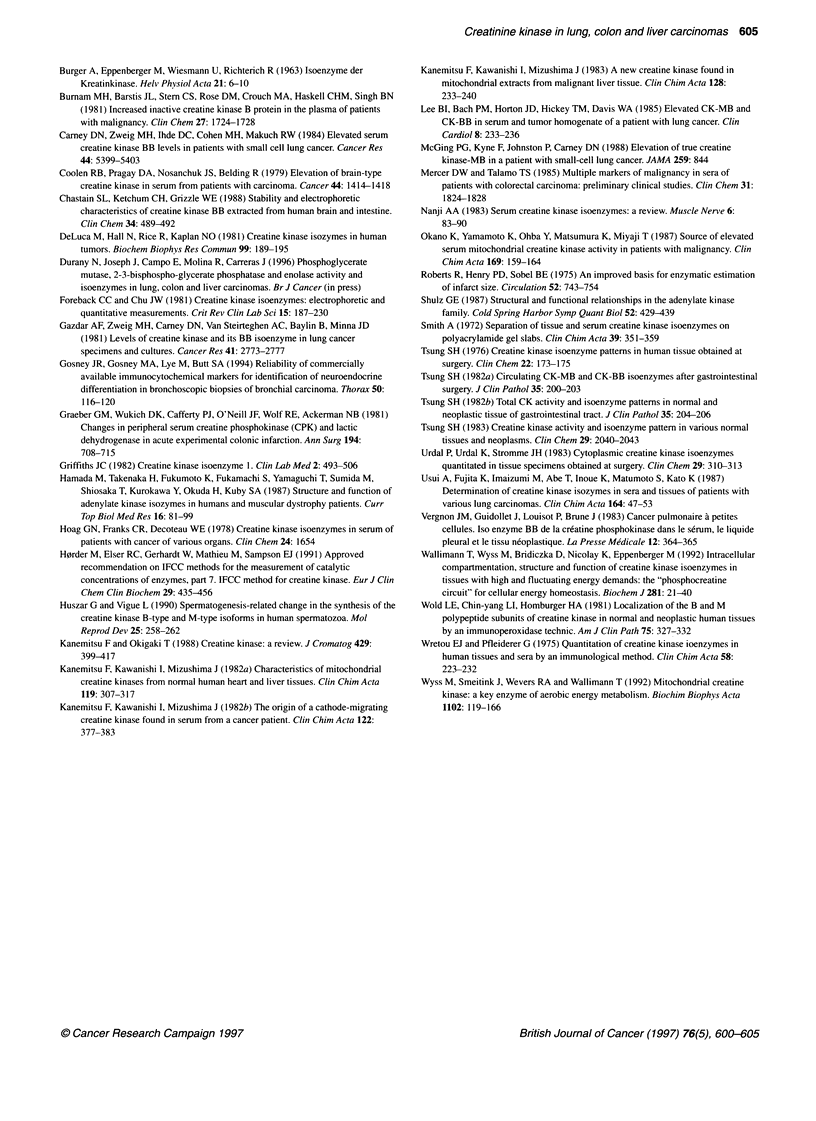# Creatine kinase activity and isoenzymes in lung, colon and liver carcinomas.

**DOI:** 10.1038/bjc.1997.432

**Published:** 1997

**Authors:** J. Joseph, A. Cardesa, J. Carreras

**Affiliations:** Unit of Biochemistry, Faculty of Medicine, University of Barcelona, Casanova, Spain.

## Abstract

**Images:**


British Journal of Cancer (1997) 76(5), 600–605
© 1997 Cancer Research Campaign

### Creatine kinase activity and isoenzymes in lung, colon and liver carcinomas

**J Joseph[1], A Cardesa[2] and J Carreras[1]**

[1]Unit of Biochemistry, Faculty of Medicine, University of Barcelona, Casanova 143, Barcelona-08036, Spain; [2]Department of Pathological Anatomy, Clínic Hospital, Villarroel 170, Barcelona-08036, Spain

**Summary** We have compared the levels of creatine kinase (CK) activity and the distribution of CK isoenzymes determined by agarose gel electrophoresis in normal colon, liver and lung tissues, and in colon, liver and lung adenocarcinomas, lung squamous cell carcinomas and lung carcinoids. Colon and lung adenocarcinomas, and squamous cell carcinomas presented lower CK activity than the normal tissues and no differences were found between hepatocarcinoma and normal liver tissue. In contrast, lung carcinoids had higher CK activity than normal lung tissue. Type BB-CK was the predominant isoenzyme in normal lung, colon and liver tissues. Type MM isoenzyme was detected in normal lung and type MB-CK was found in normal colon. In most lung tumours the CK isoenzyme electrophoretic pattern did not change. However, no type BB-CK was detected in some hepatocarcinomas, type MM-CK decreased in lung carcinoids and type MB isoenzyme was not observed in colon adenocarcinomas. It is concluded that in most tumours there is a decrease in the expression of type B- and type M-CK subunits, whereas in lung carcinoid the expression of type B-CK activity increases. Thus, the increase in type BB-CK observed in the serum of patients with lung and colon adenocarcinomas is probably due mainly to enhanced enzyme release as a result of tumour cell necrosis.

**Keywords:** creatine kinase; isoenzyme; lung; colon and liver adenocarcinoma; lung squamous cell carcinoma; lung carcinoid

Creatine kinase (ATP:creatine *N*-phosphotransferase, EC 2.7.3.2, CK) is a ubiquitous enzyme that catalyses the reversible transphosphorylation reaction between ATP and creatine, generating ADP and phosphocreatine (for a review see Bessman and Carpenter, 1985). In mammalian tissues four CK subunits are expressed, encoded by different genes: two cytosolic subunits (M-CK and B-CK) and two mitochondrial (Mt-CK) subunits, 'ubiquitous' Mt-CK (uMt-CK) and 'sarcomeric' Mt-CK (sMt-CK). In vivo, M-CK and B-CK subunits combine to give three dimeric cytosolic CK isoenzymes: type MM-, MB-, and BB-CK. MM-CK is rather specific for differentiated striated muscle (Burger et al., 1963) and mature spermatozoa (Huszar and Vigue, 1990). BB-CK is found in most adult tissues as well as in embryonic skeletal and cardiac muscle. MB-CK is detected in adult mammalian heart and in straited muscle during the developmental transition. The Mt-CK subunit forms octameric and dimeric molecules located within the mitochondrial intermembrane space (for review see Foreback and Chu, 1981; Wallimann et al, 1992; Wyss et al, 1992).

The present study was undertaken to determine the distribution of the total CK activity and isoenzymes in lung, colon and liver carcinomas as a first step to study the alterations of the expression of creatine kinase isoenzymes in neoplastic cells. Many reports have been published on the distribution of CK subunits and isoenzymes in tumours (for review see Foreback and Chu, 1981; Bais and Edwards, 1982; Griffiths, 1982; Nanji, 1983; Kanemitsu and Okigaki, 1988). However, most data have been obtained by immunohistochemical and immunoassay techniques.

*Received 21 November 1996*
*Revised 15 February 1997*
*Accepted 24 February 1997*

*Correspondence to:* J Carreras

### MATERIALS AND METHODS

### Materials

Enzymes, substrates, co-factors and biochemicals were purchased from either Boehringer (Mannheim, Germany) or Sigma (St Louis, MI, USA). CK-MB DS reaction mixture (cat. no. 1.12948) from Merck (Darmstadt, Germany) was used as source of M-CK antibodies. β-Mercaptoethanol was from Merck and bovine serum albumin was from Calbiochem (La Jolla, CA, USA). Other chemicals were reagent grade. Agar noble was obtained from Difco Laboratories (Detroit, MI, USA) and agarose gels were from Ciba-Corning (Palo Alto, CA, USA).

### Tissue samples

Tumour samples were obtained from surgical resection specimens: seven lung adenocarcinomas, five lung squamous cell carcinomas, two carcinoid tumours of the lung, ten colon adenocarcinomas and six hepatocarcinomas. Samples of normal tissue were obtained from adjacent normal tissue that had to be removed during tumour surgery.

### Tissue extraction

Tissue extracts were prepared by homogenization in three volumes (w/v) of cold Tris-buffer (20 mM *Tris*, 1 mM EDTA, 1 mM β-mercaptoethanol, pH 7.5) with a Polytron homogenizer (Lucerne, Switzerland) (position 5, 20 s). Cellular debris were removed by

**Table 1** Creatine kinase activity in human lung, colon, and liver normal tissues and tumours

| Tissue | Tumour | Case no. | | Normal tissue U g⁻¹ᵃ | Normal tissue U mg⁻¹ᵇ | Tumour U g⁻¹ᵃ | Tumour U mg⁻¹ᵇ |
|---|---|---|---|---|---|---|---|
| Lung | Adenocarcinoma | 1 | | 16 | 0.3 | 3.6 | 0.07 |
| | | 2 | | 24 | 0.4 | 10 | 0.1 |
| | | 3 | | 19 | 0.6 | 12 | 0.3 |
| | | 4 | | 6.9 | 0.26 | 4.6 | 0.15 |
| | | 5 | | 8.3 | 0.2 | 2.9 | 0.08 |
| | | 6 | | 13.8 | 0.28 | 11.1 | 0.28 |
| | | 7 | | 9.5 | 0.26 | 4.8 | 0.1 |
| | | | Mean ± s.e.m. | 13.9 ± 2.3 | 0.33 ± 0.05 | 7.0 ± 1.5 | 0.15 ± 0.04 |
| | | | Median (range) | 13.8 (6.9–24) | 0.28 (0.2–0.6) | 4.8 (2.9–12.0) | 0.1 (0.07–0.30) |
| | Squamous cell carcinoma | 1 | | 23 | 0.3 | 12 | 0.1 |
| | | 2 | | 25 | 0.3 | 4.1 | 0.1 |
| | | 3 | | 16 | 0.3 | 9.4 | 0.2 |
| | | 4 | | 12.8 | 0.2 | 2.6 | 0.05 |
| | | 5 | | 9.6 | 0.4 | 3.4 | 0.16 |
| | | | Mean ± s.e.m. | 17.3 ± 2.9 | 0.3 ± 0.03 | 6.3 ± 1.8 | 0.12 ± 0.03 |
| | | | Median (range) | 16 (9.6–25) | 0.3 (0.2–0.4) | 4.1 (2.6–12.0) | 0.1 (0.05–0.2) |
| | Carcinoid | 1 | | 21 | 0.4 | 151 | 2.6 |
| | | 2 | | 15.5 | 0.5 | 201 | 9.5 |
| | | | Mean ± s.e.m. | 18.2 ± 2.7 | 0.45 ± 0.05 | 176 ± 25 | 6.05 ± 3.45 |
| | | | Median (range) | 18.2 (15.5–21) | 0.45 (0.4–0.5) | 176 (151–201) | 6.05 (2.6–9.5) |
| Colon | Adenocarcinoma | 1 | | 73 | 1.4 | 14 | 0.3 |
| | | 2 | | 136 | 3.0 | 49 | 0.9 |
| | | 3 | | 93 | 1.8 | 47 | 0.8 |
| | | 4 | | 95 | 1.7 | 44 | 0.7 |
| | | 5 | | 132 | 4.9 | 3 | 0.07 |
| | | 6 | | 179 | 4.9 | 25 | 0.35 |
| | | 7 | | 88 | 1.3 | 113 | 2.3 |
| | | 8 | | 23 | 1.3 | 38 | 1.05 |
| | | 9 | | 68 | 1.3 | 72 | 1.4 |
| | | 10 | | 147 | 2.6 | 74 | 1.4 |
| | | | Mean ± s.e.m. | 103.4 ± 14.3 | 2.4 ± 0.45 | 47.9 ± 10.2 | 0.92 ± 0.2 |
| | | | Median (range) | 94 (23–179) | 1.75 (1.3–4.9) | 45.5 (3–113) | 0.85 (0.07–2.3) |
| Liver | Hepatocarcinoma | 1 | | 2.2 | 0.18 | 1.9 | 0.13 |
| | | 2 | | 6.6 | 0.10 | 8.0 | 0.14 |
| | | 3 | | 7.3 | 0.10 | 4.6 | 0.13 |
| | | 4 | | 5.2 | 0.11 | 4.2 | 0.11 |
| | | 5 | | 4.0 | 0.10 | 4.1 | 0.17 |
| | | 6 | | 4.7 | 0.07 | 3.9 | 0.13 |
| | | | Mean ± s.e.m. | 5.0 ± 0.75 | 0.11 ± 0.01 | 4.46 ± 0.8 | 0.13 ± 0.008 |
| | | | Median (range) | 4.9 (2.2–7.3) | 0.10 (0.07–0.18) | 4.15 (1.9–8.0) | 0.13 (0.11–0.17) |

The activity is expressed as units of activity per g of wet tissue and as units per mg of extracted protein. The contrasts are as follows: [a]lung (median and range: 15.7, 6.9–25) vs colon, $P < 0.0001$; lung (median and range: 15.7, 6.9–25) vs liver, $P < 0.001$; colon vs liver, $P < 0.001$; [b]lung (median and range: 0.3, 0.2–0.6) vs colon, $P < 0.0001$; lung (median and range: 0.3, 0.2–0.6) vs liver, $P < 0.0001$; colon vs liver, $P < 0.001$.

centrifugation (12 500 *g*, 30 min, 4°C) and the supernatants were used for the assay of CK activity and isoenzymes.

### Enzyme assays

CK activity was measured spectrophotometrically at 30°C essentially as recommended by the International Federation of Clinical Chemistry (Hørder et al., 1991). The assay is based on the formation of ATP linked to the production of NADPH via hexokinase and glucose-6-phosphate dehydrogenase. The reaction mixture contained 100 mM imidazole acetate, 2 mM EDTA, 10 mM magnesium acetate, 2 mM ADP, 5 mM AMP, 20 mM *N*-acetylcysteine, 20 mM D-glucose, 2 mM NADP, 30 mM phosphocreatine, hexokinase (3 U $ml^{-1}$), glucose-6-phosphate dehydrogenase (2 U $ml^{-1}$), pH 6.7. Enzyme activities were expressed as U $g^{-1}$ wet tissue and as U $mg^{-1}$ protein (1 unit = 1 μmol of substrate converted per min). Protein was determined by the method of Bradford (1976) using bovine serum albumin as a standard.

### Isoenzyme analysis

CK isoenzymes were separated by electrophoresis in agarose gels (Corning, cat. no 470104). Electrophoresis was performed at 4°C in Tris-sodium barbital buffer, pH 8.8 (Electra HR Buffer, cat. no. 5805), for 60 min at 95 V. CK isoenzymes were stained using a mixture containing 50 mM *Tris*, 90 mM phosphocreatine, 12 mM ADP, 60 mM magnesium chloride, 6 mM NAD, 60 mM glucose, 60 mM *N*-acetylcysteine, 15 mM AMP, 0.2 mM adenosine pentaphosphate, hexokinase (9 U $ml^{-1}$), glucose-6-phosphate dehydrogenase (7.5 U $ml^{-1}$), pH 6.7. After electrophoresis was completed,

© Cancer Research Campaign 1997

**Figure 1** Electrophoretograms of CK isoenzymes in extracts of lung normal tissue and tumours. Lane 1, human heart extract; lanes 2, 6 and 12, adenocarcinomas; lanes 4, 8 and 10, squamous cell carcinomas; lanes 14 and 16, carcinoids; lanes 3, 5, 7, 9, 11, 13, 15 and 17, corresponding normal tissues the agarose gel plate was covered with 16 ml of staining mixture (freshly prepared) containing 1% (w/v) agar gel, and incubated at 37°C for 20 min in the dark. The gels were air-dried and photographed with a Polaroid MP 4 Land Camera. The photograph was scanned at 500 nm with a Shimadzu CS-9000 densitometer. Linearity in the quantitation of bands was determined by applying different aliquots containing increasing amounts of CK. The method was found to be linear (correlation coefficient of 0.99) up to 8.5 mU of CK, with a lower detection limit of 0.6 mU of CK.

### Inhibition of CK-M subunit

Aliquots of lung (10 μl) and colon (25 μl) extracts containing 8 and 45 U $ml^{-1}$ respectively were mixed with 40 μl and 25 μl respectively of the solution containing CK-M antibodies. After 3 min of incubation at 30°C, the mixture was cooled in an ice bath, and the CK isoenzymes were determined as described above.

### Statistical analysis

Repeated measures were employed for statistical evaluation, and to compare CK activity in tumour and control tissues the Wilcoxon *t*-test was used. In order to compare CK activity levels among different tissues, the Kruskal–Wallis test was employed. The difference between groups was identified with the Mann–Whitney *U*-test. All *P*-values are two-tailed. Values are reported as mean ± s.e.m. and as median and range. Data were analysed by INSTAT statistical software.

### RESULTS

### Distribution of CK activity in lung, colon and liver normal tissues and tumours

Table 1 summarizes the levels of total CK activity in normal lung, colon and liver tissues, and in their tumours. As shown, colon is the tissue with highest CK content. When expressed as U $mg^{-1}$ of protein, the CK activity of colon extracts is about sevenfold that of lung extracts and about 20-fold that of liver extracts.

Extracts of colon and lung adenocarcinomas possess lower CK activity than the corresponding normal tissues ($P < 0.05$). Lung squamous cell carcinomas tend also to have lower CK activity than normal lung tissue, although the differences observed are not statistically significant. In contrast, the extracts of two carcinoids of the lung possess much higher CK levels than the extracts of the corresponding normal tissue. No differences have been found between hepatocarcinomas and normal liver tissue.

### Distribution of CK isoenzymes in lung normal tissue and tumours

Figure 1 shows some of the electrophoretograms of lung tumours and of the corresponding normal tissues determined by agarose gel electrophoresis.

In all 14 normal specimens studied by us, one anodic band was detected, corresponding to type BB-CK, and one or two very thin cathodic bands that migrate in a similar manner to the dimeric and octameric forms of Mt-CK (lanes 7, 9, 11 and 13) was defected. In four samples we observed an additional band in a position equivalent to that of type MM-CK (lanes 3 and 5), but type MB-CK was not detected in any specimens.

In a control experiment (not shown), no band was visualized when phosphocreatine was omitted from the staining mixture, which proved that the bands were not adenylate kinase. As shown in Figure 2, the band migrating as type MM-CK was not detected after incubation of lung extract with anti-M-CK antibodies. In contrast, the cathodic bands migrating as Mt-CK were not affected by this treatment. Thus, we conclude that BB-CK is the main CK-cytosolic isoenzyme present in normal human lung tissue, although MM-CK can be also detected.

We found that the CK isoenzyme electrophoretic patterns of extracts of adenocarcinomas (Figure 1, lanes 2, 6, and 12) and of squamous cell carcinomas of the lung (Figure 1, lanes 4, 8 and 10) did not present qualitative differences compared with extracts of the corresponding normal tissues. In contrast, in two carcinoids of the lung, we observed a change in the normal CK electrophoretic pattern. Only the band of type BB-CK was detected in the extracts

© Cancer Research Campaign 1997

of the carcinoids (Figure 1, lanes 14 and 16), whereas an additional cathodic band migrating as Mt-CK was observed in the corresponding normal tissues.

### Distribution of CK isoenzymes in colon normal tissue and tumours

In all extracts of normal colon (Figure 3, lanes 3, 5 and 7), we observed the presence of BB-CK and of two cathodic bands migrating as the dimeric and octameric forms of Mt-CK. In addition, an anodic band in a position corresponding to that of MB-CK was observed in three out of ten specimens (lanes 3 and 5). As shown in Figure 2 (lane 6), the band migrating as MB-CK was undetectable after incubation of the extract with anti-M-CK antibodies, whereas the two cathodic bands were not affected by this treatment. BB-CK was predominant in all specimens. The proportion of Mt-CK with respect to the total CK activity could not be quantified from the electrophoretograms, as in order to detect the Mt-CK bands it was necessary to apply large volumes of the extracts, over the limit of proportionality of the staining method.

As in the normal tissue, in colon adenocarcinomas (Figure 3 lanes 2, 4 and 6) BB-CK was the predominant CK isoenzyme. However, the band of MB-CK detected in some normal specimens was not present in tumoral tissue, which shows a decrease in the expression of type M-CK subunit.

**Figure 2** Effect of M-CK antibodies on the CK electrophoretic bands detected in extracts of human skeletal muscle, lung and colon. Lanes 1, 3 and 5, muscle, lung and colon extracts; lanes 2, 4 and 6, muscle, lung and colon extracts treated with M-CK antibodies

### Distribution of CK isoenzymes in liver normal tissue and tumours

As shown in Figure 3 (lanes 11, 13 and 15), we detected in six extracts of normal liver the band corresponding to BB-CK and two cathodic bands corresponding to Mt-CK. In addition, we detected a faint band slightly ahead of MM-CK, which in a control experiment (not shown) was proved to correspond to adenylate kinase not inhibited by the AMP present in the staining mixture. It is known that liver adenylate kinase is more resistant to inhibition than the other adenylate kinase isoenzymes (Hamada et al, 1987; Schulz et al, 1987). No MM-CK was detected in any specimen.

Most of the extracts of hepatocarcinomas analysed by us showed the same CK electrophoretic pattern as that of normal liver tissue. However, BB-CK was not detected in two of them (Figure 3, lane 14).

**Figure 3** Electrophoretograms of CK isoenzymes in extracts of human liver and colon normal tissues and tumours. Colon specimens are shown in **A**: lanes 2, 4 and 6, adenocarcinomas; lanes 3, 5 and 7, normal tissues. Liver specimens are shown in **B**: lanes 10, 12 and 14, hepatocarcinomas; lanes 11, 13 and 15, normal tissues. Lanes 1 and 9, human heart extracts. Lanes 8 and 16, human brain extracts (cortex)

© Cancer Research Campaign 1997

### DISCUSSION

Our data show that colon and lung adenocarcinomas and squamous cell carcinomas of the lung possess lower CK activity than the corresponding normal tissues. In contrast, lung carcinoids have higher CK levels and no difference exists between CK levels in hepatocarcinomas and in normal liver tissue. These results agree with other published results on colon carcinomas (Tsung, 1982*b*, 1983) and lung carcinomas (Coolen et al, 1979; Gazdar et al, 1981; Tsung, 1983). Most data obtained by immunological techniques (Wretou and Pfleiderer, 1975; Usui et al, 1987), ion-exchange chromatography (Roberts et al, 1975; Tsung 1976, 1983; Vergnon, 1983) and electrophoresis (Allard and Cabrol, 1970; Smith, 1972; Coolen et al, 1979) have shown that in normal lung BB-CK is the predominant isoenzyme. MM-CK is found in a much lower proportion and MB-CK is present at very low levels. Only one report (Roberts et al, 1975) detected higher levels of MM-CK than of BB-CK. Our results confirm that BB-CK is the major CK cytosolic isoenzyme in normal lung tissue, although MM-CK is also present at much lower levels. The absence of MB-CK in extracts that possess MM- and BB-CK suggests that type M- and type B-CK subunits are expressed in different lung cell types.

Using immunological techniques, some authors (Wold et al, 1981; Gosney et al, 1994) found lower expression of BB-CK in small-cell lung carcinomas than in other lung carcinomas. However, other authors (Gazdar et al, 1981; Carney et al, 1984; Usui et al, 1987) have reported that the levels of BB-CK in small-cell lung carcinomas are much greater than those of normal lung, and that the BB-CK levels in non-small-cell lung carcinomas are lower than those of normal lung (Gazdar et al, 1981; Usui et al, 1987). In all types of lung carcinoma (except one case of small-cell carcinoma), MB-CK and MM-CK levels were found by Usui et al (1987) to be low and similar to those of normal lung. Using ion-exchange chromatography (Tsung, 1983) and by electrophoretic analysis (Coolen et al, 1979; De Luca et al, 1981; Gazdar et al, 1981; Tsung, 1983; Lee et al, 1985; McGing et al, 1988), it has also been found that BB-CK is the predominant CK isoenzyme in several types of lung carcinoma. Our results show that, whereas lung adenocarcinomas and squamous cell carcinomas possess BB- and MM-CK isoenzymes in a proportion similar to those of the normal lung tissue, the proportion of BB-CK increases in carcinoids of the lung.

It has been shown by electrophoresis (Griffiths, 1982; Tsung, 1982*a*, 1982*b*, 1983; Urdal et al, 1983; Chastain et al, 1988), ion-exchange chromatography (Tsung, 1976, 1982*a*, 1982*b*, 1983) and immunoinhibition (Chastain et al, 1988) that human colon possesses mainly type BB-CK. BB-CK has been found to be predominant also in colon adenocarcinomas (Coolen et al, 1979; Tsung 1982*b*, 1983). However, Graeber et al (1981) reported significant amounts of MM-CK and MB-CK in normal colon, and Tsung (1982*b*, 1983) indicated that colon malignant tissue had a higher proportion of MM-CK than normal tissue. The conflicting results can be explained by the confusion created by Mt-CK and BB-CK. Mt-CK, which is more abundant in colon tumours than in normal colon tissue (Okano et al, 1987; McGing et al, 1988), can contaminate the MM-CK band. Post-mortem changes in BB-CK can produce a band with an electrophoretic mobility similar to that of MB-CK (Chastain et al, 1988). Our results show that BB-CK is the major CK isoenzyme in normal colon tissue and in colon adenocarcinoma, and that MB-CK, which is found in low proportion in normal tissue, is not detectable in colon tumours.

The distribution of CK isoenzymes in human liver has been determined by immunotitration (Wretou and Pfleiderer, 1975), ion-exchange chromatography (Roberts et al, 1975; Wretou and Pfleiderer, 1975; Tsung, 1976, 1983) and electrophoresis (Allard and Cabrol, 1970; Smith, 1972; Griffiths, 1982; Tsung, 1983). Some studies have reported only type BB-CK, but others have detected predominantly MM-CK. In two human liver tumours carried in athymic mice, DeLuca et al (1981) showed a high proportion of BB-CK and a low concentration of MM-CK and Mt-CK. The discrepancies probably result from confusion created by Mt-CK. Liver mitochondrial extracts have two cathodic Mt-CK forms that can be converted into a new form at the MM-CK position after long-term storage (Kanemitsu, 1982*a*, 1982*b*, 1983). Moreover, liver Mt-CK is readily released (Urdal et al, 1983). Our results confirm that BB-CK is the predominant CK isoenzyme in both normal liver tissue and hepatocarcinoma, although two of six tumours presented a large decrease in BB-CK levels.

In summary, the results herein presented demonstrate that, in contrast to phosphoglycerate mutase and enolase activities (Durany et al, 1997), CK activity does not increase in most lung, colon and liver carcinomas. One exception is the carcinoid of the lung which, as has been reported to occur in small-cell lung carcinoma, presents higher CK activity than the normal tissue. The results also show that in most tumours there is a decrease in the expression of type M-CK subunit that is present at very low levels in normal lung, and colon tissues.

Normal human serum contains MM-CK almost exclusively and it has been suggested that serum BB-CK could be used as a tumour marker (Foreback and Chu, 1981; Bais and Edwards, 1982; Griffiths, 1982; Nanji, 1983; Kanemitsu and Okigaki, 1988). Serum BB-CK increases in patients with small-cell lung carcinoma, which has higher BB-CK levels than the normal lung tissue. But serum BB-CK has also been found increased in patients with squamous cell carcinoma and adenocarcinoma of the lung and in patients with late-stage colon adenocarcinoma (Hoag et al, 1978; Coolen et al, 1979; Burnam et al, 1981; Griffiths, 1982; Mercer and Talamo, 1985; Usui et al, 1987; Arenas et al, 1989). As these tumours have lower BB-CK levels than the normal tissues, we conclude that the increase observed in serum BB-CK levels is probably mainly due to enhanced enzyme release from tumour cell necrosis.

### ACKNOWLEDGEMENTS

This work was supported by FIS (Grant 93/0573), by Generalitat de Catalunya, grant GR0-1036 project (PL 93/1359) and by Hospital Clínic de Barcelona (Grant 1993). We are grateful to J Ojuel and to J Parra for advice on statistical analysis.

### REFERENCES

Allard and Cabrol D (1970) Etude eletrophoretique des isozymes de la creatine phosphokinase dans les tissus de l'homme et du lapin. *Path Biol* **18**: 847–850

Arenas J, Diaz AE, Alcaide MJ, Santos I, Martinez A and Culebras JM (1989) Serum CK-BB as a tumor marker in patients with carcinoma confirmed histologically. *Clin Chim Acta* **182**: 183–194

Bais R and Edwards JB (1982) Creatine kinase. *Crit Rev Clin Lab Sci* **16**: 291–335

Bessman SP and Carpenter CL (1985) The creatine-creatine phosphate energy shuttle. *Ann Rev Biochem* **54**: 831–862

Bradford MMA (1976) A rapid and sensitive method for the quantitation of microgram quantities of protein utilizing the principle of protein-dye binding. *Anal Biochem* **72**: 248–254

© Cancer Research Campaign 1997

Burger A, Eppenberger M, Wiesmann U, Richterich R (1963) Isoenzyme der Kreatinkinase. *Helv Physiol Acta* **21**: 6–10

Burnam MH, Barstis JL, Stern CS, Rose DM, Crouch MA, Haskell CHM, Singh BN (1981) Increased inactive creatine kinase B protein in the plasma of patients with malignancy. *Clin Chem* **27**: 1724–1728

Carney DN, Zweig MH, Ihde DC, Cohen MH, Makuch RW (1984) Elevated serum creatine kinase BB levels in patients with small cell lung cancer. *Cancer Res* **44**: 5399–5403

Coolen RB, Pragay DA, Nosanchuk JS, Belding R (1979) Elevation of brain-type creatine kinase in serum from patients with carcinoma. *Cancer* **44**: 1414–1418

Chastain SL, Ketchum CH, Grizzle WE (1988) Stability and electrophoretic characteristics of creatine kinase BB extracted from human brain and intestine. *Clin Chem* **34**: 489–492

DeLuca M, Hall N, Rice R, Kaplan NO (1981) Creatine kinase isozymes in human tumors. *Biochem Biophys Res Commun* **99**: 189–195

Durany N, Joseph J, Campo E, Molina R, Carreras J (1996) Phosphoglycerate mutase, 2-3-bisphospho-glycerate phosphatase and enolase activity and isoenzymes in lung, colon and liver carcinomas. *Br J Cancer* (in press)

Foreback CC and Chu JW (1981) Creatine kinase isoenzymes: electrophoretic and quantitative measurements. *Crit Rev Clin Lab Sci* **15**: 187–230

Gazdar AF, Zweig MH, Carney DN, Van Steirteghen AC, Baylin B, Minna JD (1981) Levels of creatine kinase and its BB isoenzyme in lung cancer specimens and cultures. *Cancer Res* **41**: 2773–2777

Gosney JR, Gosney MA, Lye M, Butt SA (1994) Reliability of commercially available immunocytochemical markers for identification of neuroendocrine differentiation in bronchoscopic biopsies of bronchial carcinoma. *Thorax* **50**: 116–120

Graeber GM, Wukich DK, Cafferty PJ, O'Neill JF, Wolf RE, Ackerman NB (1981) Changes in peripheral serum creatine phosphokinase (CPK) and lactic dehydrogenase in acute experimental colonic infarction. *Ann Surg* **194**: 708–715

Griffiths JC (1982) Creatine kinase isoenzyme 1. *Clin Lab Med* **2**: 493–506

Hamada M, Takenaka H, Fukumoto K, Fukamachi S, Yamaguchi T, Sumida M, Shiosaka T, Kurokawa Y, Okuda H, Kuby SA (1987) Structure and function of adenylate kinase isozymes in humans and muscular dystrophy patients. *Curr Top Biol Med Res* **16**: 81–99

Hoag GN, Franks CR, Decoteau WE (1978) Creatine kinase isoenzymes in serum of patients with cancer of various organs. *Clin Chem* **24**: 1654

Hørder M, Elser RC, Gerhardt W, Mathieu M, Sampson EJ (1991) Approved recommendation on IFCC methods for the measurement of catalytic concentrations of enzymes, part 7. IFCC method for creatine kinase. *Eur J Clin Chem Clin Biochem* **29**: 435–456

Huszar G and Vigue L (1990) Spermatogenesis-related change in the synthesis of the creatine kinase B-type and M-type isoforms in human spermatozoa. *Mol Reprod Dev* **25**: 258–262

Kanemitsu F and Okigaki T (1988) Creatine kinase: a review. *J Cromatog* **429**: 399–417

Kanemitsu F, Kawanishi I, Mizushima J (1982*a*) Characteristics of mitochondrial creatine kinases from normal human heart and liver tissues. *Clin Chim Acta* **119**: 307–317

Kanemitsu F, Kawanishi I, Mizushima J (1982*b*) The origin of a cathode-migrating creatine kinase found in serum from a cancer patient. *Clin Chim Acta* **122**: 377–383

Kanemitsu F, Kawanishi I, Mizushima J (1983) A new creatine kinase found in mitochondrial extracts from malignant liver tissue. *Clin Chim Acta* **128**: 233–240

Lee BI, Bach PM, Horton JD, Hickey TM, Davis WA (1985) Elevated CK-MB and CK-BB in serum and tumor homogenate of a patient with lung cancer. *Clin Cardiol* **8**: 233–236

McGing PG, Kyne F, Johnston P, Carney DN (1988) Elevation of true creatine kinase-MB in a patient with small-cell lung cancer. *JAMA* **259**: 844

Mercer DW and Talamo TS (1985) Multiple markers of malignancy in sera of patients with colorectal carcinoma: preliminary clinical studies. *Clin Chem* **31**: 1824–1828

Nanji AA (1983) Serum creatine kinase isoenzymes: a review. *Muscle Nerve* **6**: 83–90

Okano K, Yamamoto K, Ohba Y, Matsumura K, Miyaji T (1987) Source of elevated serum mitochondrial creatine kinase activity in patients with malignancy. *Clin Chim Acta* **169**: 159–164

Roberts R, Henry PD, Sobel BE (1975) An improved basis for enzymatic estimation of infarct size. *Circulation* **52**: 743–754

Shulz GE (1987) Structural and functional relationships in the adenylate kinase family. *Cold Spring Harbor Symp Quant Biol* **52**: 429–439

Smith A (1972) Separation of tissue and serum creatine kinase isoenzymes on polyacrylamide gel slabs. *Clin Chim Acta* **39**: 351–359

Tsung SH (1976) Creatine kinase isoenzyme patterns in human tissue obtained at surgery. *Clin Chem* **22**: 173–175

Tsung SH (1982*a*) Circulating CK-MB and CK-BB isoenzymes after gastrointestinal surgery. *J Clin Pathol* **35**: 200–203

Tsung SH (1982*b*) Total CK activity and isoenzyme patterns in normal and neoplastic tissue of gastrointestinal tract. *J Clin Pathol* **35**: 204–206

Tsung SH (1983) Creatine kinase activity and isoenzyme pattern in various normal tissues and neoplasms. *Clin Chem* **29**: 2040–2043

Urdal P, Urdal K, Stromme JH (1983) Cytoplasmic creatine kinase isoenzymes quantitated in tissue specimens obtained at surgery. *Clin Chem* **29**: 310–313

Usui A, Fujita K, Imaizumi M, Abe T, Inoue K, Matumoto S, Kato K (1987) Determination of creatine kinase isozymes in sera and tissues of patients with various lung carcinomas. *Clin Chim Acta* **164**: 47–53

Vergnon JM, Guidollet J, Louisot P, Brune J (1983) Cancer pulmonaire à petites cellules. Iso enzyme BB de la créatine phosphokinase dans le sérum, le liquide pleural et le tissu néoplastique. *La Presse Médicale* **12**: 364–365

Wallimann T, Wyss M, Bridiczka D, Nicolay K, Eppenberger M (1992) Intracellular compartmentation, structure and function of creatine kinase isoenzymes in tissues with high and fluctuating energy demands: the "phosphocreatine circuit" for cellular energy homeostasis. *Biochem J* **281**: 21–40

Wold LE, Chin-yang LI, Homburger HA (1981) Localization of the B and M polypeptide subunits of creatine kinase in normal and neoplastic human tissues by an immunoperoxidase technic. *Am J Clin Path* **75**: 327–332

Wretou EJ and Pfleiderer G (1975) Quantitation of creatine kinase ioenzymes in human tissues and sera by an immunological method. *Clin Chim Acta* **58**: 223–232

Wyss M, Smeitink J, Wevers RA and Wallimann T (1992) Mitochondrial creatine kinase: a key enzyme of aerobic energy metabolism. *Biochim Biophys Acta* **1102**: 119–166

© Cancer Research Campaign 1997